# One case of spinal bulbar muscular atrophy misdiagnosed as polymyositis: Case report

**DOI:** 10.1097/MD.0000000000039169

**Published:** 2024-09-27

**Authors:** Yv-sen Mu, Jia-yi Yao, Fang Li

**Affiliations:** a Graduate School of Hebei North University, Zhangjiakou, Hebei, China; b Department of Traditional Chinese Medicine, Jitang College, North China University of Technology, Tangshan, Hebei, China; c Department of Rheumatism and Immunology, Hebei General Hospital, Shijiazhuang, Hebei, China.

**Keywords:** spinal bulbar muscular atrophy

## Abstract

**Rationale:**

Spinal bulbar muscular atrophy (SBMA) is a rare X-linked recessive motor neuron degenerative disease. Due to the lack of specificity in its early clinical manifestations, SBMA is easily misdiagnosed. Herein, we present a case in which SBMA was misdiagnosed as polymyositis.

**Patient concerns:**

A 58-year-old patient began to develop symptoms of limb weakness 20 years ago and was admitted to the Second Hospital of Hebei Medical University 10 years ago without special treatment. Two years ago, the above symptoms worsened and he was admitted to Peking Union Medical College Hospital. The patient was misdiagnosed as polymyositis. According to the gene mutation characteristics of SBMA, the patient was diagnosed with SBMA.

**Diagnoses:**

The result of the Kennedy gene test was positive, and the patient was diagnosed with Kennedy disease.

**Interventions:**

After the diagnosis of SBMA, the patient was given symptomatic treatment to alleviate the condition.

**Outcomes:**

Conservative treatment after discharge was requested. It is recommended that patients avoid bucking to prevent complications.

**Lessons:**

This is a case of milder SBMA being misdiagnosed as polymyositis. For patients with weak limbs, the possibility of SBMA should be considered.

## 1. Introduction

**Figure 1.**
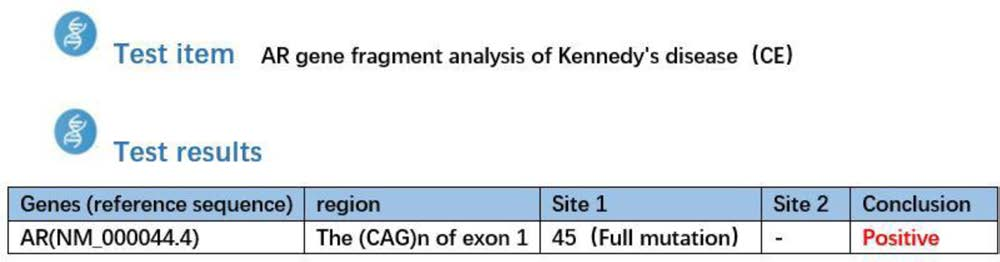
AR genetic testing results.

Spinal bulbar muscular atrophy (SBMA), also known as Kennedy Disease, was first reported by American physician William R. Kenndey in 1968.^[1]^ This disease is an X-linked recessive degenerative disease of the nervous system. The pathogenic gene is the abnormal amplification of CAG repeat sequence of androgen receptor (AR) gene exon 1 on chromosome Xq11-12, which belongs to polyglutamine disease. So the diagnosis of the disease relies heavily on genetic testing.^[2]^ As with other X-linked genetic disorders, the disease is more common in adult males and mainly occurs between the ages of 30 and 50. Women are mostly carriers of the disease-causing gene, with a reported incidence of about 1 to 2.5 per 100,000.^[3]^ Although it is very rare, this disease has brought great pain to patients, so we should be more conscious in clinical work to diagnose and differentiate this disease.^[4]^ This patient, a middle-aged male with relatively mild symptoms, was misdiagnosed as polymyositis 20 years ago. It was not diagnosed as SBMA until our hospital detected 45 CAG repeats of AR gene exon 1 through genetic testing, which was consistent with the genetic mutation characteristics of SBMA. We hope to use this case as a clinical reference to provide some experience for the diagnosis and treatment of this disease (Fig. 1).

## 2. Case report

### 2.1. Case description

A 58-year-old male patient was admitted to the hospital on March 27, 2023, mainly due to limb weakness for 20 years, which worsened for more than 1 week. More than 10 years ago, the above symptoms aggravated and the patient was treated in the Second Hospital of Hebei Medical University. The relevant examinations such as muscle biopsy did not find obvious abnormalities and no treatment was given. Two years ago, the above symptoms aggravated again, it was difficult to squat down and stand up, and it was difficult to go upstairs. The patient was treated with tacrolimus, methotrexate, and tofacitinib, but no significant improvement was observed. One week ago, the patient’s symptoms were aggravated again and he was admitted to our hospital as “polymyositis unknown.” Physical examination after admission showed that the tongue sulcus was deepened, jaw tremor, limb muscle strength was slightly weak, and no muscle atrophy was found.

### 2.2. Diagnostic methods

Biochemical examination after admission, urinalysis + quantification of urinary sediment, 5 items of lymphocyte immune analysis, antinuclear antibody, blood routine, 5 items of coagulation, 8 items of preoperative, 6 items of thyroid function, all items of male tumor, 12 items of cytokine detection, stool analysis, chest CT, abdominal color Doppler ultrasound, heart color Doppler ultrasound, anti-dsdna antibody, vasculitis screening, antinuclear antibody spectrum, thigh MRI, electromyography, 5 hormones, myositis antibody spectrum, and gene detection.

Laboratory tests showed creatine kinase 861.7U/L, CK-MB35.5 U/L, myoglobin 179 ng/mL, creatinine 48.5 μmol/L, total cholesterol 6.27 mmol/L, triglyceride 2.55 mmol/L, and immunoglobulin G7.82 g/L. Urinalysis + urine sediment quantification: protein: ±, specific gravity: 1.033, urobilinogen: 1+, lymphocyte immunoassay: total T lymphocytes (CD3+) 78.92%, helper/inducer T cells (CD3 + CD4+) 54.84%, inhibitory/cytotoxic T cells (CD3 + CD8+) 20.61%, CD4+/CD8 + 2.66, NK cells (CD3-CD16 + CD56)11.88%, antinuclear antibody negative. There were no obvious abnormalities in routine blood test, 8 of 5 coagulation tests, 6 of thyroid function tests, all of male tumor tests, 12 of cytokine tests, and stool analysis. Chest CT showed pulmonary nodules, emphysema, multiple lymph nodes, and elastofibroma. There were no specific signs in abdominal ultrasound and cardiac ultrasound. After analysis of the condition, the patient had atypical inflammatory myopathy, and the previous treatment with glucocorticoids and immunosuppressive agents was not effective. Therefore, the myositis antibody spectrum was further improved, neuroelectromyography was performed, and muscle biopsy was performed if necessary to determine the cause. Rheumatology and immunology diseases were excluded. Anti-dsdna antibody was negative, and vasculitis screening showed no abnormality in antinuclear antibody spectrum. MRI of the thigh suggested that there were multiple abnormal signals in bilateral thigh muscles, pelvic and buttock muscles, which was considered as a possibility of polymyositis. Electromyography showed extensive neuronal damage and multiple peripheral nerve damage. The patient had extensive neuronal damage, multiple peripheral nerve damage, jaw tremor, and elevated muscle enzymes. The patient’s symptoms may be related to motor neuron damage, and the patient was suspected of motor neuron disease. Five hormones were further improved to clarify the etiology. Five hormone tests: luteinizing hormone 14.84 mIU/mL, follicle stimulating hormone 5.75 mIU/mL, prolactin 18.98 ng/mL, estradiol 65.32 pg/mL, testosterone 9.28 ng/mL, myositis antibody spectrum was negative. The CAG repeat number of AR gene exon 1 was 45, which was in the full range of mutation. It was consistent with the gene mutation characteristics of Kennedy disease.

### 2.3. Treatment and results

**Figure 2.**
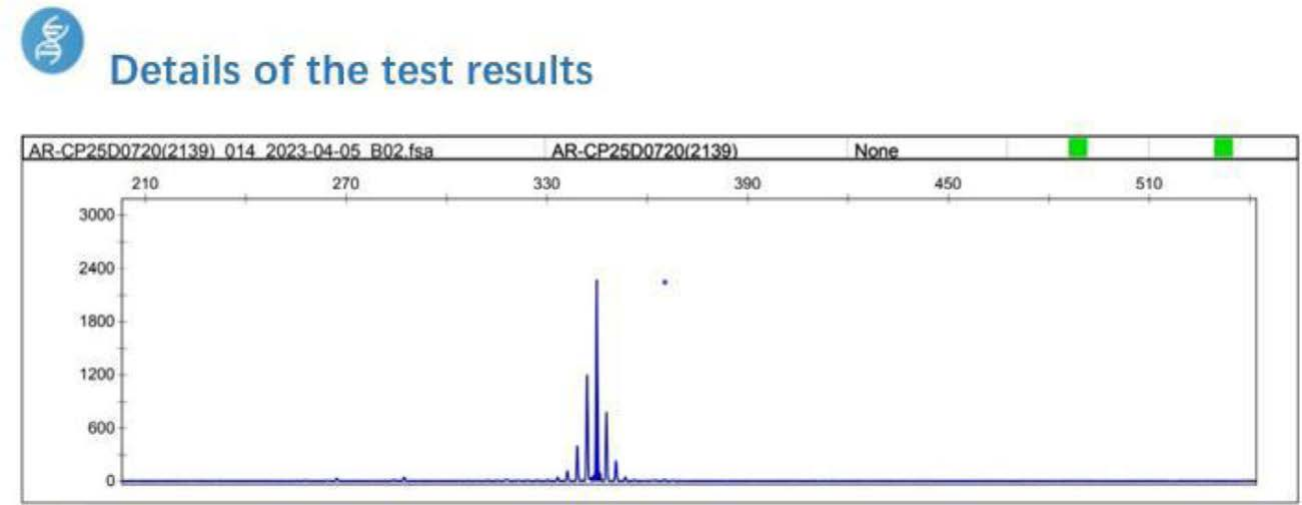
Details of the detection results.

The result of Kennedy gene test was positive, and the patient was diagnosed with Kennedy disease. The patient’s vital signs were stable (Fig. 2).

Conservative treatment after discharge was requested. It is recommended that patients avoid bucking, prevent complications, and further treatment in superior hospitals.

## 3. Discussion

SBMA is an X-linked recessive neurodegenerative disease. SBMA mainly affects motor neurons in the brain stem and spinal cord, but also includes sensory nerves, skeletal muscles, and endocrine disorders outside the nervous system. The clinical features of this disease are mainly male onset in adulthood, characterized by progressive proximal limb muscle weakness, atrophy, and true bulbar palsy. It may be accompanied by gynecomastia, testicular dysplasia, and even infertility. As the disease continues to develop, it may present with clinical manifestations such as weakness and atrophy of the distal limbs muscles.^[5]^ Movement disorders include both typical and atypical limb weakness. In this case, the patient had jaw tremor, slight weakness of limb muscle strength, no muscle atrophy, and elevated creatine kinase, which was consistent with movement disorder. At the same time, patients may also be accompanied by varying degrees of hypesthesia and endocrine abnormalities. Androgen receptor insensitivity can be manifested as gynecomastia, decreased sexual function, testicular atrophy, infertility, etc. In addition, there may also be abnormal glucose and fat metabolism.

The above symptoms of this patient are not typical.^[6]^ In addition, most of the symptoms of female carriers are asymptomatic, and even if symptoms occur, the degree is mild, which can only be manifested as fasciculation, mild limb weakness, muscle cramps, or simply elevated muscle enzymes.^[7]^ At present, there is no effective treatment for Kennedy disease, but it generally has little effect on life expectancy. Therefore, the focus of treatment should be to maintain the individual’s muscle function, delay the disease process, and improve the quality of life.^[8]^

With respect to genetics, if only the father is SBMA patient, the probability that the father will pass the defective gene to his son is zero, and the daughter is a 100% carrier. If only the mother is a carrier, there is a 50% chance that the son will have SBM and a 50% chance that the daughter will carry the disease-causing gene. (Carriers generally do not develop the disease, and if they do, symptoms are relatively mild.)^[9]^ Female carriers have low levels of testosterone, which prevents them from binding to and activating the mutant androgen receptor, thus preventing it from entering the nucleus to function, keeping the mutation in the androgen receptor protein harmless. Therefore, female carriers are generally asymptomatic.^[10]^

The clinical diagnosis of KD requires a combination of medical history, clinical examination, neuroelectro-physiological manifestations, and genetic testing results. (1) Blood test: serum creatine kinase and lactate dehydrogenase may be slightly or significantly increased, and sex hormone levels (testosterone, progesterone, follicle stimulating hormone, luteinizing hormone) may also be abnormal. Hyperlipemia and impaired glucose tolerance may occur. (2) Electromyography: sensory nerve conduction may show decreased amplitude of action potential and slow conduction velocity. (3) Biopsy: muscle biopsy: mainly manifested as neurogenic damage, sometimes combined with myogenic damage. Nerve biopsy: large myelinated fibers are reduced, a small number of fibers are demyelinated, and Schwann cells are degenerated. (4) Genetic testing: the number of CAG repeats in AR gene is the gold standard for the diagnosis of Kennedy disease. In 2011, the European Federation of Neuroscience guidelines defined the number of CAG repeats ≥35 as the basis for the diagnosis of SBMA. Similar to other polyglutamine diseases, SBMA shows the phenomenon of “genetic premature appearance,” in which the number of repeat copies increases in the process of passage, leading to the advance of the onset time and the aggravation of clinical symptoms.^[11]^ Item 1.2.4 was fulfilled in this patient, so the diagnosis of SBMA was confirmed.

There is no effective treatment for the disease at present. The treatment of Kennedy disease includes symptomatic treatment and etiological treatment. (1) Symptomatic treatment: at present, it is to relieve the clinical symptoms and improve the quality of life of patients. For example, patients with painful spasms are treated with anti-spasmolytic and analgesic drugs; patients with malnutrition caused by dysphagia can be treated with percutaneous endoscopic gastrostomy. Patients with respiratory dysfunction can use mechanical ventilation to improve symptoms. However, for gynecomastia and sexual dysfunction, conventional androgen replacement therapy may aggravate the disease progression. (2) Treatment of the cause: at present, there is still a lack of effective treatment for the cause of the disease, and potential therapeutic drugs are under study. Leuprolide is a luteinizing hormone-releasing hormone analogue, which can inhibit the secretion of gonadotropin, reduce the deposition of abnormal AR protein in the nucleus, and delay the progression of the disease.^[12]^ But another study said clinical trials of the androgen drug leuprolide did not consistently show significant efficacy, but leuprolide was effective as a treatment for dysphagia in subsequent clinical trials in Japan, leading to its approval in Japan but not elsewhere. According to animal studies, the administration of testosterone and its analogues may worsen motor neuron disease. Some new drugs, such as heat shock protein inducer, histone deacetylase and ASC-J9, have shown initial effects in animal experiments.^[13]^

The disease usually progresses slowly, the average life span is normal or slightly shortened, and the cause of death is often pneumonia and respiratory failure. Literature reports that the 10-year survival rate of the disease is 82%, and the median survival age is 65 years.^[14]^

## 4. Conclusion

Here we describe a patient with Kennedy disease, whose misdiagnosis several years ago highlighted the necessity and difficulty of differential diagnosis of such patients. Genetic testing is the key to the diagnosis of Kennedy disease. In our case, our patient presented typical clinical symptoms. The current diagnostic gold standard of SBMA is undoubtedly genetic testing, but in actual clinical work, the disease is relatively rare, and the price of genetic testing is relatively expensive, resulting in poor compliance of patients, so we should pay more attention to its clinical manifestations. Although the disease is not common, when the patient has limb weakness and other symptoms, it cautions us to perform biochemical, electromyography, muscle biopsy, genetic, and other tests to help exclude or confirm the diagnosis of SBMA.

## Author contributions

**Conceptualization:** Yv-sen Mu, Jia-yi Yao, Fang Li.

**Data curation:** Yv-sen Mu, Jia-yi Yao.

**Formal analysis:** Fang Li.

**Writing – original draft:** Yv-sen Mu, Jia-yi Yao.

**Writing – review & editing:** Fang Li.
